# Non-HDL-C and age-stratified mortality risk in the US general population: a population-based cohort study

**DOI:** 10.3389/fnut.2025.1591705

**Published:** 2025-06-13

**Authors:** Zhiqing Fu, Wei Zhang, Shan Li

**Affiliations:** Department of Cardiology, The Second Medical Center and National Clinical Research Center for Geriatric Diseases, Chinese PLA General Hospital, Beijing, China

**Keywords:** non-HDL cholesterol, mortality, age, cardiovascular disease, NHANES data

## Abstract

**Introduction:**

Non-high-density lipoprotein cholesterol (non-HDL-C) is a well-established residual causal risk factor for the progression of atherosclerotic cardiovascular disease. However, studies of large, broadly generalizable populations are lacking, and the effect of non-HDL-C on all-cause and cause-specific mortality, particularly in different age groups, remains uncertain.

**Methods:**

We conducted a population-based cohort study using data from the National Health and Nutrition Examination Survey from 1999 to 2018. Participants were divided into six groups according to non-HDL-C levels (≤100, 101–130, 131–160, 161–190, 191–220, >220 mg/dL). Multivariable Cox proportional hazards models were used to calculate hazard ratios (HR) and corresponding 95% confidence intervals (CI). Restricted cubic spline curves and subgroup analysis were also performed to further explore the association between non-HDL-C and mortality.

**Results:**

Of 51,252 individuals (mean age 48.1 ± 19.2 years), 7,605 (14.8%) died during follow-up. Both low and high non-HDL-C levels were significantly associated with increased risk of all-cause and cause-specific mortality, suggesting a U-shaped association. Thresholds of 156, 142, 162, and 152 mg/dL were identified for all-cause, cardiovascular, cancer, and other-cause mortality, respectively. We observed significant interactions between non-HDL-C and age for all-cause and cardiovascular mortality (P interaction<0.05 for each). The association of high non-HDL-C (>220 mg/dL) with all-cause and cardiovascular mortality was strongest in adults aged <50 years (HR, 1.51 [1.09–2.08] and 1.97 [1.07–3.12], respectively), intermediate in adults aged 50 to 69 years, and weakest in adults aged ≥70 years.

**Conclusion:**

Non-HDL-C was U-shaped associated with all-cause and cause-specific mortality in the US general population. However, in younger adults (<50 years), the higher the non-HDL-C, the higher the risk of cardiovascular and all-cause mortality. These observations support clear public health messaging and strict adherence to primary prevention strategies for atherosclerosis in younger adults. This has important implications for the development of age-specific interventions to reduce mortality associated with non-HDL-C levels.

## Introduction

Non-high-density lipoprotein cholesterol (non-HDL-C) has been recognized as a significant factor in residual atherosclerotic cardiovascular disease (ASCVD) risk among patients on statin therapy with well-controlled low-density lipoprotein cholesterol (LDL-C) levels (1–3). Therefore, most international guidelines recommend non-HDL-C as a secondary target for dyslipidemia management (4–7). Elevated levels of non-HDL-C consistently indicate an increased risk of future atherosclerotic cardiovascular events across diverse populations worldwide (8–10). In addition, numerous randomized controlled trials of novel lipid-lowering agents have demonstrated that reducing non-HDL-C levels correlates with a reduced risk of future atherosclerotic cardiovascular events (11–13).

While it is widely accepted that high levels of non-HDL-C are associated with an increased risk of mortality, it is uncertain whether low levels are associated with a decreased risk. Studies investigating the relationship between non-HDL-C levels and the risk of all-cause and cardiovascular mortality have yielded mixed results, with some showing a straightforward positive association (higher mortality with increasing non-HDL-C levels) and others showing a U-shaped association (14). Most of these studies have been conducted in specific patient populations, such as those with coronary heart disease (15), diabetes (16), hypertension (17), and chronic kidney disease (CKD) (18), with few studies focusing on the general population. Furthermore, although a sub-analysis of a consortium database found that higher baseline non-HDL-C levels have a greater effect on the incidence of cardiovascular events in younger individuals (<45 years) than in older individuals (≥60 years) (3), the effect of non-HDL-C on age-specific mortality has not been extensively reported. Consequently, the association between non-HDL-C levels and the risk of all-cause and cause-specific mortality in the general population at different ages remains unclear. In addition, the specific non-HDL-C concentration associated with the lowest risk of mortality has not been definitively established.

In this study of the US general population, we aimed to examine the association between non-HDL-C levels and the risk of all-cause and cause-specific mortality in adults of different ages. In addition, we sought to identify the non-HDL-C level associated with the lowest risk of mortality in individuals.

## Materials and methods

### Study population

This study used data from the National Health and Nutrition Examination Survey (NHANES), a nationally representative survey conducted by the Centers for Disease Control and Prevention (CDC) to assess the health and nutrition status of the non-institutionalized U. S. population (19). Data from 10 survey cycles conducted between 1999 and 2018 were included. Of the 101,316 participants, 51,252 individuals were ultimately included in the analysis after excluding those who were <18 years of age, pregnant, had missing follow-up data, were missing total cholesterol (TC) or high-density lipoprotein cholesterol (HDL-C), or had extremely high non-HDL-C levels (>400 mg/dL, *n* = 20, see Supplementary Figure S1).

### Exposure variables

Fasting blood samples were collected and analyzed for TC, triglycerides, and HDL-C using the Roche Cobas 6,000 (c501 module) analyzer. LDL-C was calculated using the Friedewald equation. Fasting status was defined as time since last meal ≥8 h at the time of blood sampling. Non-HDL-C levels were derived from TC minus HDL-C and categorized into six groups: ≤100, 101–130, 131–160, 161–190, 191–220, and >220 mg/dL, according to previous studies (18, 20).

### Outcomes

Mortality data were obtained from the National Center for Health Statistics and included all-cause and cause-specific mortality, with cause-specific categories including cardiovascular death, cancer death, and other-cause death. Mortality events were followed from enrollment through December 31, 2019. Cause-specific mortality was defined based on the recorded NCHS underlying classification of death (UCOD). Cardiovascular death was defined as death due to cardiovascular disease (054–068 and 070). Cancer death was defined as death due to malignant neoplasm (019–043). Other-cause death was defined as death from causes other than cardiovascular disease and cancer. UCOD codes were also obtained for the subcategories of cause-specific mortality, including heart disease (054–068), cerebrovascular disease (070), respiratory disease (076–078 and 082–086), Alzheimer’s disease (052), diabetes mellitus (046), and nephrotic disease (097–101). The subcategories of cancer mortality were defined by the type of tumor suffered.

### Covariates data

Data were collected on age, sex, ethnicity, smoking status, education level, marital status, time since last meal, statin use, blood pressure, body mass index (BMI), and medical conditions using a computer-assisted interview system. Blood pressure was measured 3 times consecutively after resting quietly for 5 min, and height, weight, and waist circumference were measured at the Mobile Examination Center (MEC) in disposable medical gowns and using a uniform methodology. Major medical conditions were determined by physician report of a specific disease or use of prescription medications associated with the disease. ASCVD included coronary artery disease and ischemic stroke. More information is available at www.cdc.gov/nchs/nhanes.

### Statistical analysis

The data were presented as mean values with standard deviation (SD), the median with interquartile ranges (IQRs), or frequencies with percentages, as appropriate. Comparisons of the differences between groups were made with one-way ANOVA, chi-square tests, or Kruskal-Wallis H-tests by non-HDL-C groups. Cox proportional hazards models were used to estimate hazard ratios (HRs) and 95% confidence intervals (CIs) of all-cause and cause-specific mortality for non-HDL-C on categorical or continuous scales. The proportional hazards assumption was tested and confirmed by Schoenfeld residuals and log-time plots. Two models were fitted. Model I, unadjusted. Model II, adjusted for age, sex, ethnicity, systolic blood pressure, BMI, HDL-C, ASCVD, diabetes mellitus, chronic obstructive pulmonary disease (COPD), renal dysfunction, cancer, fasting status. Covariate data were more than 95% complete, and missing values (4.9% for systolic blood pressure, 1.7% for BMI) were imputed using chained equation multiple imputation (Nimputation = 5) to minimize sample size reduction. No imputation was performed for missing data because rates of missingness for all covariates included in the models was <1%. The multiplicative interaction of continuous non-HDL-C and potential effect modifiers on mortality endpoints was examined using the Wald test. The dose–response association between non-HDL-C and mortality was explored using restricted cubic spline (RCS) analysis. Three knots were chosen according to the Akaike information criterion to balance best fit and overfitting. If the association exhibited nonlinearity, the threshold value was estimated by trying all possible values, choosing the threshold point with the highest likelihood. Subsequently, a two-piece wise Cox proportional risk model was employed on both sides of the inflection point to investigate the association between non-HDL-C and the risk of all-cause and cause-specific mortality. Bootstrap resampling was used to determine confidence intervals for thresholds. Furthermore, the subgroup analysis includes age (<50, 50–69 and ≥70 years), sex (male, female), ethnicity (White, non-White), BMI (<25 or ≥25 kg/m^2^), smoking (yes or no), HDL-C (<40 or ≥40 mg/dL), ASCVD (yes or no), statin use (yes or no). To assess the possibility that the association of non-HDL-C with outcomes in individuals may differ across age strata, we tested for effect modification between age and non-HDL-C on outcomes, using multiplicative interaction terms of age × non-HDL-C in all multivariable models. Next, the adjusted association between non-HDL-C and outcomes was compared across 3 age strata (<50, 50–69, and ≥70 years old). RCSs were generated to visually represent the continuous association of non-HDL-C with outcomes across the 3 age strata. These analyses were adjusted for the variables in model 2.

Sensitivity analyses were performed to test the robustness of the results. First, additional adjustments for triglycerides to account for the strong association between triglycerides and residual ASCVD risk. Second, additional adjustments for socioeconomic factors such as education level, marital status, and poverty income ratio (PIR). Third, additional adjustments for anthropometric measures reflecting abdominal obesity (waist circumference) to account for the stronger association between abdominal obesity and cardiometabolic risk than central obesity as indicated by body mass index. Fourth, individuals with 1 year and <2 years of follow-up were excluded to minimize potential bias for reverse causality. Fifth, the association was examined between non-HDL-C and risk of all-cause and cause-specific mortality in individuals with or without statin use.

All statistical analyses were performed using R version 4.3.0 (R Foundation for Statistical Computing, Vienna, Austria), and a 2-sided *p* < 0.05 was considered statistically significant.

## Results

### Baseline characteristics

Finally, 51,252 individuals (mean age 48.1 ± 19.2 years), weighted to represent 200 million US adults, were included in this analysis. During a median follow-up of 9.7 years (IQR 5.0–14.1), 7,605 (14.8%) all-cause deaths, 2,393 (4.7%) cardiovascular deaths, 1,647 (3.2%) cancer deaths, and 3,565 (7.0%) other-cause deaths were documented. Table 1 shows baseline characteristics by non-HDL-C level. Higher non-HDL-C levels were associated with older age, male sex, white race, current smoking, higher SBP and BMI, and lower statin use (Table 1).

**Table 1:** Baseline characteristics of individuals by non-HDL-C category.

Characteristics	Non-HDL-C, mg/dL						*p*-value
	≤100	101–130	131–160	161–190	191–220	>220	
Unweighted							
*N* (%)	8,232 (16.1)	13,812 (26.9)	14,003 (27.3)	8,938 (17.4)	4,143 (8.1)	2,124 (4.1)	
All-cause mortality, *n* (%)	1,113 (13.5)	1948 (14.1)	2036 (14.5)	1,425 (15.9)	672 (16.2)	411 (19.4)	<0.0001
Cardiovascular mortality, *n* (%)	375 (4.6)	590 (4.3)	621 (4.4)	448 (5.0)	227 (5.5)	132 (6.2)	<0.0001
Cancer mortality, *n* (%)	205 (2.5)	435 (3.1)	462 (3.3)	323 (3.6)	131 (3.2)	91 (4.3)	<0.0001
Other-cause mortality, *n* (%)	533 (6.5)	923 (6.7)	953 (6.8)	654 (7.3)	314 (7.6)	188 (8.9)	<0.0001
Weighted							
*N* (%)	31,210,612 (14.9)	55,345,232 (26.5)	58,512,724 (28.0)	37,620,943 (18.0)	17,256,425 (8.3)	9,154,434 (4.4)	
Age, years	41.8 ± 19.9	44.4 ± 18.2	47.3 ± 16.7	48.8 ± 15.6	49.1 ± 14.8	50.0 ± 14.0	<0.0001
Male, %	45.6	45.4	49.2	52.3	55.5	55.5	<0.0001
Ethnicity, %							<0.0001
Non-Hispanic White	64.7	67.1	69.4	70.3	70.1	72.7	
Non-Hispanic Black	15.2	11.9	9.9	8.8	7.9	6.9	
Hispanic	12.9	13.7	13.9	14.8	15	13.6	
Other	7.2	7.3	6.8	6.1	7.1	6.7	
Systolic blood pressure, mmHg	118.2 ± 17.1	120.4 ± 17.2	122.8 ± 17.3	124.6 ± 17.7	126.2 ± 18.0	128.8 ± 18.4	<0.0001
Body mass index, kg/m^2^	26.5 ± 6.7	27.9 ± 7.0	29.3 ± 6.8	29.7 ± 6.2	29.9 ± 5.9	29.9 ± 5.6	<0.0001
Smoking status, %							<0.0001
Never smoker	60.8	57.4	55.4	53.7	49.1	43.4	
Former smoker	20.2	22.5	24.6	24.5	26	28.2	
Current smoker	19	20.1	20	21.8	25	28.4	
Total cholesterol, mg/dL	142.7 ± 21.1	170.8 ± 18.1	196.3 ± 17.1	222.6 ± 16.3	249.8 ± 15.7	293.6 ± 41.6	<0.0001
HDL cholesterol, mg/dL	58.5 ± 18.2	55.7 ± 16.4	52.4 ± 15.3	49.6 ± 14.4	47.7 ± 13.5	45.0 ± 12.5	<0.0001
LDL cholesterol, mg/dL	69.1 ± 13.3	95.3 ± 12.5	119.1 ± 13.9	142.7 ± 15.2	166.6 ± 16.8	203.4 ± 31.2	<0.0001
Non-HDL-C, mg/dL	83.6 ± 12.5	115.1 ± 8.6	143.9 ± 8.6	173.0 ± 8.5	202.1 ± 8.6	246.5 ± 27.1	<0.001
Triglycerides, mg/dL	65.0 (49.0, 89.0)	87.0 (64.0, 121.0)	110.0 (80.0, 152.0)	139.0 (101.0, 193.0)	166.0 (121.0, 244.0)	225.0 (160.0, 342.6)	<0.0001
Fasting status†, %	54.5	54.5	54.3	52.9	52.8	52.3	0.204
Statin use, %	22.5	18.9	13	8.7	6.8	7.9	<0.0001
ASCVD, %	8.7	5.9	4.6	4.4	4.5	6.1	<0.0001
Diabetes mellitus, %	12	9.1	7.3	7.2	7.7	10.5	<0.0001
COPD, %	5.5	5.7	5.7	5.7	6.8	8.2	0.0026
Renal dysfunction, %	8	6.1	6	6	6	6.4	<0.0001
Cancer, %	8.6	9.1	9.3	9.2	9.1	8.6	0.8569
Education level, %							<0.0001
Under high school	24.1	25.7	26.9	28.7	30.7	31.8	
High school graduate	23.1	22.2	23.2	23.1	24.9	24.8	
Above high school	52.8	52.1	49.9	48.1	44.4	43.3	
Marital status							<0.0001
Married/cohabiting	49.1	55	60.6	63	62.5	61.3	
Separated/divorced/widowed	19.3	20.7	21.8	22.9	24.5	26.9	
Never married/unknown	31.6	24.3	17.6	14.1	12.9	11.8	
Poverty income ratio (PIR)^#^	2.8 ± 1.7	2.9 ± 1.6	3.1 ± 1.6	3.1 ± 1.6	2.9 ± 1.6	2.9 ± 1.6	<0.0001

†Fasting status, fasting time ≥ 8 h. #PIR is calculated by dividing family income by family size, year, and geographic location, as measured by the Department of Health and Human Services. ASCVD, atherosclerotic cardiovascular disease. HDL, high-density lipoprotein. LDL, low-density lipoprotein. Non-HDL-C, non-HDL Cholesterol. COPD, chronic obstructive pulmonary disease.

### Relationships of non-HDL-C with all-cause and cause-specific mortality

**Figure 1:**
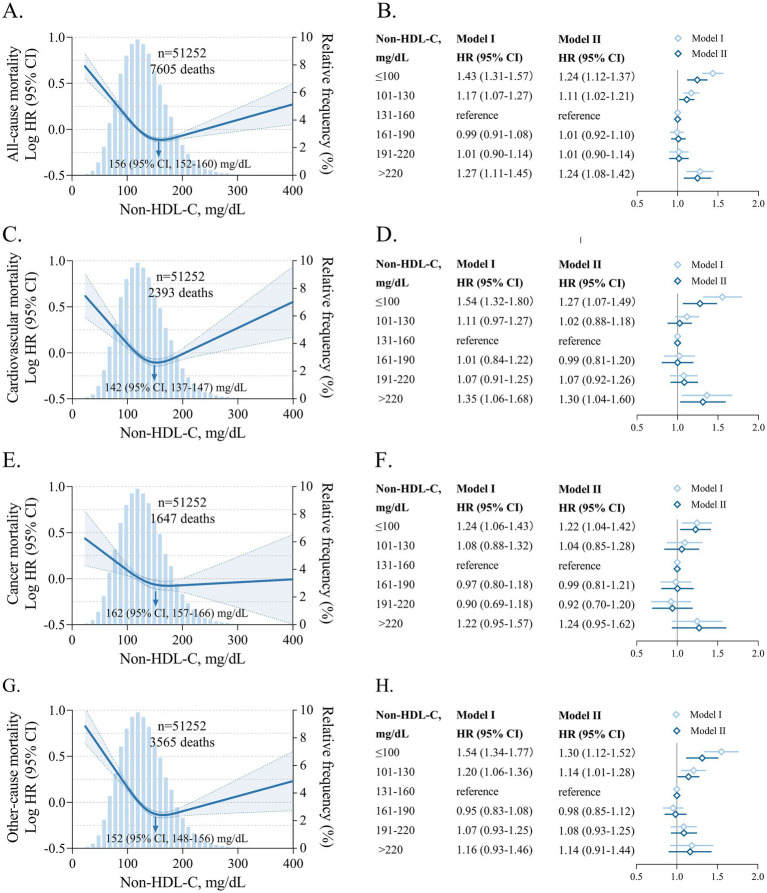
All-cause and cause-specific mortality risks by non-HDL-C on continuous and categorical scales. Associations between non-HDL-C and all-cause **(A,B)**, cause-specific mortality **(C–H)** on continuous and categorical scales. HRs (solid lines) and 95% CIs (shaded areas) are based on restricted cubic splines with Cox model adjusted for age, sex, ethnicity, systolic blood pressure, body mass index, HDL cholesterol, ASCVD, diabetes mellitus, COPD, renal dysfunction, cancer, fasting status. The blue histograms show the distribution of non-HDL-C in the population. Model I, unadjusted. Model II, adjusted for age, sex, ethnicity, systolic blood pressure, body mass index, HDL cholesterol, ASCVD, diabetes mellitus, COPD, renal dysfunction, cancer, fasting status. HDL, high-density lipoprotein. Non-HDL-C, non-high-density lipoprotein cholesterol. ASCVD, atherosclerotic cardiovascular disease. COPD, chronic obstructive pulmonary disease.

The association between non-HDL-C levels on a continuous scale and risk of all-cause mortality was U-shaped; low and high levels of non-HDL-C were associated with increased risk of all-cause mortality, and the optimal range was 131–160 mg/dL (Figure 1). Compared with individuals with non-HDL-C concentrations of 131–160 mg/dL, the multivariable-adjusted HR for all-cause mortality was 1.24 (95% CI 1.12 to 1.37) for individuals with non-HDL-C concentrations ≤100 mg/dL and 1.24 (95% CI 1.08 to 1.42) for non-HDL-C concentrations >220 mg/dL. The non-HDL-C level associated with the lowest risk of all-cause mortality was 156 (95% CI 152 to 160) mg/dL.

Similar U-shaped associations were seen for non-HDL-C and risk of cause-specific mortality. Compared with individuals with non-HDL-C concentrations of 131–160 mg/dL, the multivariable-adjusted HRs for cardiovascular, cancer, and other-cause mortality were 1.27 (95% CI 1.07 to 1.49), 1.22 (95% CI 1.04 to 1.42), 1.30 (95% CI 1.12 to 1.52) for individuals with non-HDL-C concentrations ≤100 mg/dL and 1.30 (1.04 to 1.60), 1.24 (0.95 to 1.62), 1.14 (0.91 to 1.44) for non-HDL-C concentrations >220 mg/dL. The non-HDL-C concentrations associated with the lowest risk of cardiovascular, cancer, and other-cause mortality were 142 (95% CI 137 to 147), 162 (95% CI 157 to 166), and 152 (95% CI 148 to 156) mg/dL, respectively.

### Subgroups analysis of the risk of all-cause and cause-specific mortality

**Figure 2:**
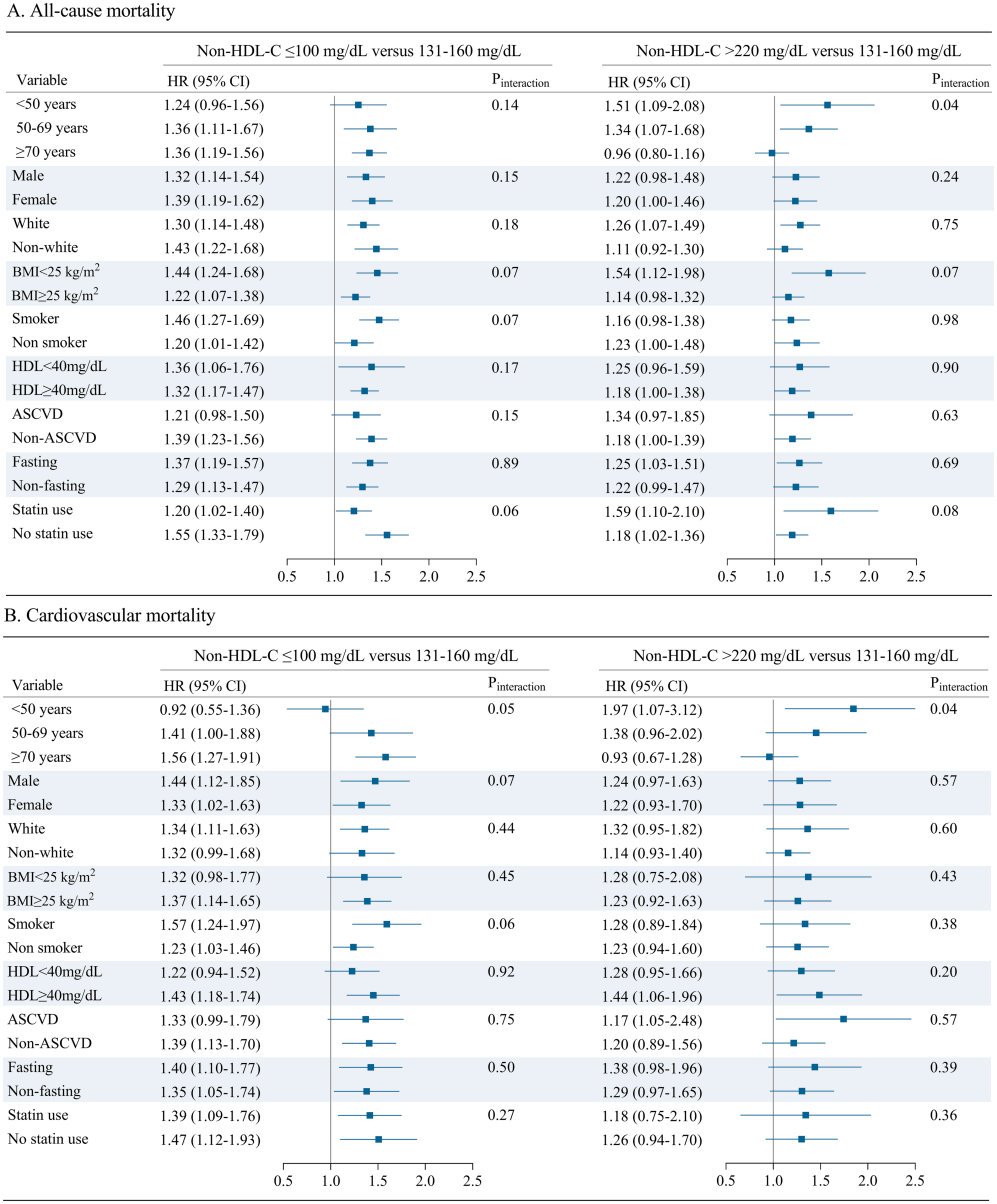
Multivariable adjusted hazard ratios with 95% confidence intervals for all-cause and cardiovascular mortality all-cause **(A)** and cardiovascular mortality **(B)**. for the highest and lowest level of non-HDL-C group. The highest level group (>220 mg/dL) vs. the reference group (131–160 mg/dL) of non-HDL-C and the lowest level group (≤100 mg/dL) vs. the reference group of (131–160 mg/dL) of non-HDL-C, respectively. *p* for interaction was examined by including a two-factor interaction term between the examined non-HDL-C and each covariate in the Cox proportional hazards regression. Analyses were adjusted for age, sex, ethnicity, systolic blood pressure, body mass index, HDL cholesterol, ASCVD, diabetes mellitus, COPD, renal dysfunction, cancer, fasting status. BMI, body mass index. HDL, high-density lipoprotein. Non-HDL-C, non-high-density lipoprotein cholesterol. ASCVD, atherosclerotic cardiovascular disease. COPD, chronic obstructive pulmonary disease.

Stratified analysis by age, sex, ethnicity, BMI, smoking status, HDL-C, ASCVD, fasting status, and statin use further examined the association of the highest and lowest non-HDL-C groups with all-cause and cause-specific mortality (Figure 2 and Supplementary Figure S2). In age-stratified analyses, the positive association between the highest non-HDL-C group and all-cause and cardiovascular mortality generally decreased with age, whereas the inverse association between the lowest non-HDL-C group and all-cause and cardiovascular mortality did not change significantly (*p* interaction <0.05, Figure 2). This phenomenon was not observed for cancer and other-cause mortality. No significant differences were observed in the other subgroups analyzed, with the exception of age.

### Effect-modifying association of non-HDL-C with all-cause and cardiovascular mortality by age

**Figure 3:**
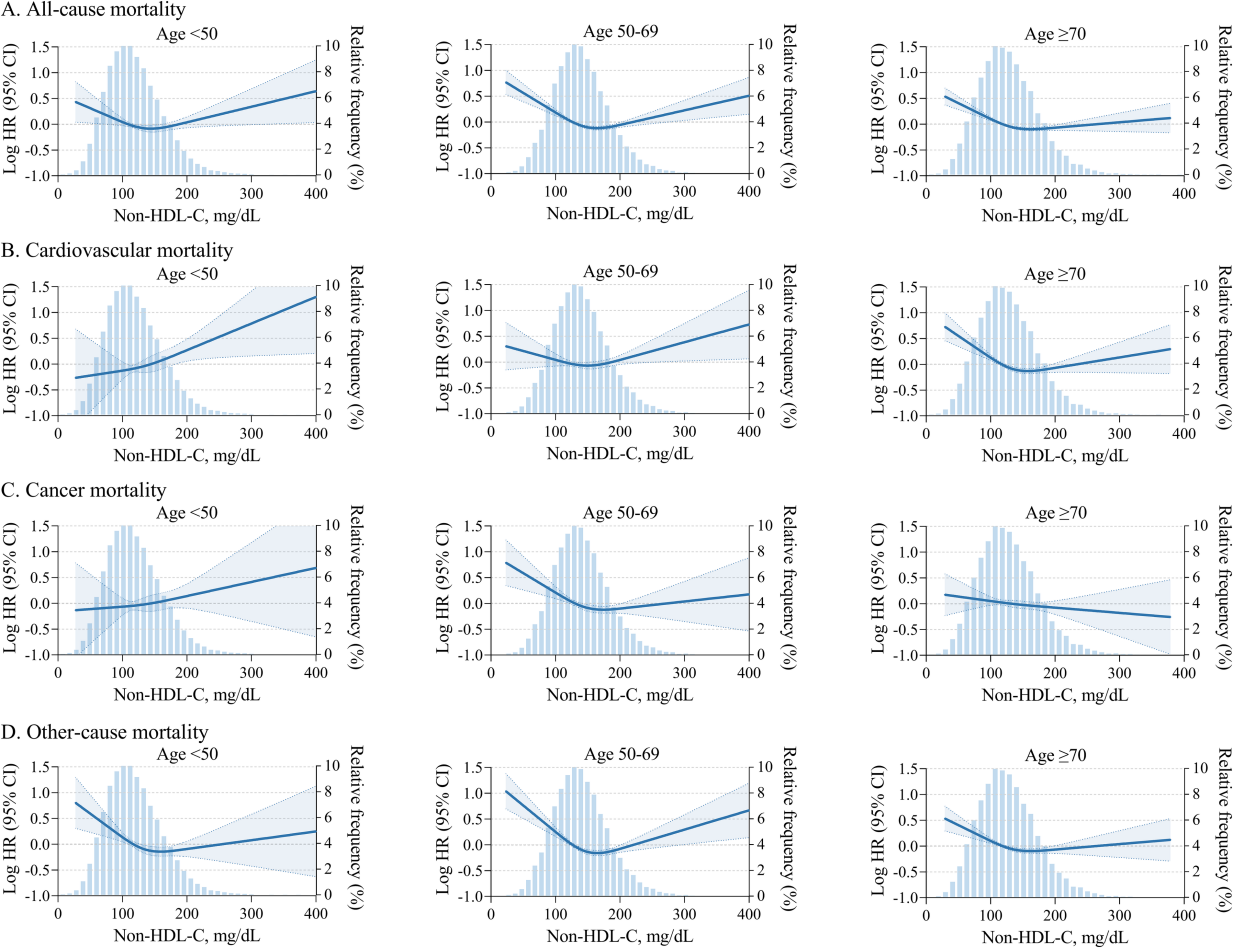
All-cause and cause-specific mortality risks by age and non-HDL-C. Adjusted hazards ratio for all-cause mortality **(A)**, cardiovascular mortality **(B)**, cancer mortality **(C)**, and other-cause mortality **(D)** by continuous non-HDL-C and ages<50, 50 to 69, and ≥70 years. The blue histograms show the distribution of non-HDL-C in the population. Adjustment variables include age, sex, ethnicity, systolic blood pressure, body mass index, HDL cholesterol, ASCVD, diabetes mellitus, COPD, renal dysfunction, cancer, fasting status. Non-HDL-C, non-high-density lipoprotein cholesterol. HDL, high-density lipoprotein. ASCVD, atherosclerotic cardiovascular disease. COPD, chronic obstructive pulmonary disease.

We observed effect modification by age on the associations between non-HDL-C and the outcomes of all-cause and cardiovascular mortality. We then evaluated the associations between continuous non-HDL-C and the outcomes by age strata of <50, 50 to 69, and ≥70 years using adjusted cubic splines (Figure 3). Although higher non-HDL-C was associated with greater odds of all-cause and cardiovascular mortality across all age strata, the slope of the odds ratio spline for all-cause and cardiovascular mortality was steeper in those <50 years than in the older groups (Figure 3). Furthermore, the association of the highest non-HDL-C group (>220 mg/dL) with all-cause and cardiovascular mortality was strongest in adults <50 years (HR, 1.51 [1.09–2.08] and 1.97 [1.07–3. 12], respectively), intermediate in adults 50 to 69 years (HR, 1.34 [1.07–1.68] and 1.38 [0.96–2.02]), and weakest in adults ≥70 years (HR, 0.96 [0.80–1.16] and 0.93 [0.67–1.28], Figure 2). In contrast, the association of low non-HDL-C (≤100 mg/dL) with cardiovascular mortality was strongest in adults >70 years (HR, 1.56 [1.27–1.91]), intermediate in adults 51 to 70 years (HR, 1.41 [1.00–1.88]), and weakest in adults <50 years (HR, 0.92 [0.55–1.36]). The youngest group (<50 years) generally had a positive linear association with cardiovascular mortality.

### Sensitivity analyses

Several sensitivity analyses were performed. After additional adjustment for triglycerides (Supplementary Figure S3), additional adjustment for socioeconomic factors (Supplementary Figure S4), and additional adjustment for waist circumference (Supplementary Figure S5), the results did not change substantially. The results were similar when individuals with 1 year or less than 2 years of follow-up were excluded from the analysis (Supplementary Figures S6, S7). Finally, the results were generally consistent among statin users and non-users, except that cancer mortality was not significantly associated with non-HDL-C among statin users (Supplementary Figure S8). For the cause-specific mortality subcategories, U-shaped associations were generally found. Exceptions were respiratory diseases and accidents, which showed linear associations (Supplementary Figure S9). We also analysed the other age subgroups (< 55, 55 −65 and ≥ 65 years; < 60, 60 −79 and ≥ 80 years), with generally consistent results (Supplementary Figures S10, S11).

## Discussion

This analysis has demonstrated several important findings regarding the effect of non-HDL-C levels on the risk of all-cause and cause-specific mortality. Firstly, our study demonstrated a U-shaped relationship, indicating that both low and high non-HDL-C levels were associated with an increased risk of all-cause and cause-specific mortality in the US general population. Secondly, we identified specific thresholds of non-HDL-C levels that were associated with the lowest risk of all-cause, cardiovascular, cancer, and other-cause mortality, providing valuable clinical guidance. Thirdly, our results revealed a significant interaction between age and non-HDL-C levels, with the association between high non-HDL-C levels and all-cause, cardiovascular mortality being stronger in younger individuals compared with older individuals. Notably, the youngest group (<50 years) showed a positive linear association between non-HDL-C and cardiovascular mortality. These observations shed light on the complex relationship between non-HDL-C and mortality, with implications for risk stratification and targeted interventions across different age groups.

Previous cohort studies have shown inconsistent results regarding the association between non-HDL-C and all-cause mortality (21–25). Our study found that non-HDL-C levels above or below a certain threshold are associated with increased mortality in the U. S. population, possibly due to the role of extremely high levels in accelerating atherosclerosis (26, 27) and the impact of low levels on health status (28–31). As with LCL-C, the association between low non-HDL-C levels and an increased risk of all-cause mortality could be explained by reverse causality. It has been hypothesized that debilitation and disease can lead to a decline in cholesterol levels (31, 32). In this study, individuals with the lowest non-HDL-C levels were found to be more likely to have multiple comorbidities. In addition, higher HDL-C levels have been associated with increased mortality, possibly related to genetic variation and HDL particle function (17). The U-shaped association between non-HDL-C levels and mortality may have similarities to the obesity paradox, but further research is needed to clarify the mechanisms driving this association.

Our study showed that the lowest non-HDL-C risk thresholds for all-cause and cardiovascular mortality in the general population were 156 and 142 mg/dL (range, 131–160), respectively. This is somewhat different from the results of previous studies. For example, one study found that the lowest risk non-HDL-C thresholds for all-cause and cardiovascular mortality in the general population were 158 and 190 mg/dL, respectively (17), and another study in patients with CKD reported that the optimal range of non-HDL-C concentrations for risk of all-cause and cardiovascular mortality was 116.2 to 143.9 mg/dL (18). Estimates of non-HDL-C thresholds associated with lowest risk of mortality may differ because of differences in study populations and ages. Our threshold estimates are based on a large multiethnic sample population and are more credible than other assessments.

One study that examined the interaction between non-HDL-C and age found that high baseline non-HDL-C levels had a greater impact on cardiovascular disease incidence in younger patients (<45 years) than in older patients (≥60 years) (3). However, there was no corresponding report on mortality risk. In our study, age was found to significantly modify the association of non-HDL-C with all-cause and cardiovascular mortality. High baseline non-HDL-C levels had a greater impact on all-cause and cardiovascular mortality in younger individuals (<50 years), and in particular, non-HDL-C levels were positively and linearly associated with cardiovascular death. This finding highlights the importance of lipid-lowering therapy for primary prevention of cardiovascular disease in younger individuals. The stronger positive associations and lower optimal ranges we observed in the younger age groups may be due to the positive association between age and non-HDL-C levels (33), as well as higher non-HDL-C concentrations and higher proportions of cardiovascular mortality in younger Americans (34). In contrast, we observed a weaker effect size for high non-HDL-C in older adults, similar to previous findings for TC and LDL-C (35, 36). This may reflect the competing risks of death due to multiple chronic conditions, frailty, or other factors in the elderly.

In our study, we also found U-shaped associations between non-HDL-C and mortality from cancer and other causes, with the lowest non-HDL-C thresholds for mortality associated with these diseases falling between 131 and 160 mg/dL. These findings are consistent with those of Hovsepyan G, who found that very high non-HDL-C was associated with higher all-cause and cancer-specific mortality in postmenopausal women with obesity-related cancers (37). In addition, higher HDL-C levels were inversely associated with all-cause and cancer-specific mortality (37). While high non-HDL-C is significantly associated with increased incidence of certain cancers (38–40), the relationship with cancer prognosis remains uncertain. Our study also showed associations between non-HDL-C and mortality in diabetic and nephropathic patients, consistent with previous findings (16, 18). In addition, a similar association has been shown between non-HDL-C and Alzheimer’s disease due to ApoE4 disruption of brain lipid homeostasis and energy transduction (41–43). However, we did not find an association between non-HDL-C and respiratory mortality. Interestingly, higher non-HDL-C levels were associated with fewer accidental deaths, possibly due to reduced activity in obese individuals.

Our findings may have important implications for understanding what constitutes a “normal and healthy” level of non-HDL-C in the general population, especially if the focus is not limited to ASCVD. The finding that the lowest risk of all-cause mortality was associated with a non-HDL-C level of 156 mg/dL (4.0 mmol/L) suggests that in individuals at otherwise low risk of ASCVD, a non-HDL-C level around this level may not be inherently dangerous. However, any increase in non-HDL-C was associated with an increased risk of death from ASCVD in younger adults (<50 years). Taken together, these results underscore the importance of taking age into account when deciding whether to initiate lipid-lowering therapy, rather than starting treatment on the basis of a moderate rise in non-HDL-C alone.

### Strengths and limitations

Our study, the largest NHANES study to date, comprehensively reported the relationship between non-HDL-C and all-cause and disease-specific mortality in the US general population and, in particular, found that the relationship between non-HDL-C and all-cause and cardiovascular mortality was largely influenced by age. Higher non-HDL-C had a greater impact on all-cause and cardiovascular mortality in younger patients than in older patients. This has important implications for future lipid-lowering programs in the primary prevention of cardiovascular disease in the general population.

There are several limitations in this study. Firstly, as an observational study, we cannot establish a causal relationship between non-HDL-C and mortality because of potential confounding factors. Secondly, this study only assessed the prognostic value of the baseline non-HDL-C and did not investigate the association between changes in non-HDL-C over time and mortality. Thirdly, we only had information on lipid-lowering treatment at baseline and cannot exclude that the results may have been influenced by individuals starting or stopping lipid-lowering treatment during follow-up. Some results were corrected for regression dilution bias to visualize the potential underestimation of effect estimates. Fourthly, some comorbidities are self-reported, which may introduce potential biases. Finally, the study population consisted primarily of the general population in the US, so caution should be exercised when extrapolating the findings to other ethnicities.

## Conclusion

This study demonstrates a significant association between non-HDL-C levels and all-cause and cardiovascular mortality across age groups. The findings highlight the importance of monitoring non-HDL-C levels in assessing mortality risk, particularly in the context of cardiovascular health. Further research is needed to establish causality and to understand the implications of these findings in diverse populations.

## Data Availability

Publicly available datasets were analyzed in this study. This data can be found here: https://www.cdc.gov/nchs/nhanes/.
