# Supplementary material for: Non-HDL-C and age-stratified mortality risk in the US general population: a population-based cohort study
Source: Front Nutr. 2025 Jun 13;12:1591705. doi: 10.3389/fnut.2025.1591705 (PMC12202456; doi:10.3389/fnut.2025.1591705)

Supplementary Materials

**Non-HDL-C and Age-Stratified Mortality Risk in the US General Population: A Population-Based Cohort Study**

Zhiqing Fu, MD^1^, Wei Zhang, MD^1^, Shan Li, MD^1^ *

^1^Department of cardiology, the Second Medical Center & National Clinical Research Center for Geriatric Diseases, Chinese PLA General Hospital, Beijing, China.

**Contents**

[Figure S1. Flow chart of the study design 3](#_Toc6726)

[Figure S2. Subgroup analyses of cancer and other-cause mortality risks in the ≤100mg/dL, >200mg/dL vs. 131-160mg/dL non-HDL-C group (n=51252) 4](#_Toc28417)

[Figure S3. All-cause and cause-specific mortality for continuous non-HDLC with additional adjustment for triglycerides (n=51252) 5](#_Toc15289)

[Figure S4. All-cause and cause-specific mortality for continuous non-HDL-C with additional adjustment for socioeconomic factors (n=51252) 6](#_Toc4680)

[Figure S5. All-cause and cause-specific mortality for continuous non-HDL-C with additional adjustment for waist circumference (n=51252) 7](#_Toc16423)

[Figure S6. All-cause and cause-specific mortality for continuous non-HDL-C with exclusion of individuals with 1 year of follow-up (n=50791) 8](#_Toc10963)

[Figure S7. All-cause and cause-specific mortality for continuous non-HDL-C with exclusion of individuals with <2 years of follow-up (n=47864) 9](#_Toc13096)

[Figure S8. All-cause and cause-specific mortality risks in individuals with or without statin use 10](#_Toc4493)

[Figure S9. Subcategory of cause-specific mortality risks by non-HDL-C on continuous scales 11](#_Toc24289)

[Figure S10. All-cause and cause-specific mortality risks by age (<55, 55-65, and ≥65 years) and non-HDL-C 12](#_Toc4425)

[Figure S11. All-cause and cause-specific mortality risks by age (<60, 60-80, and ≥80 years) and non-HDL-C 13](#_Toc28439)

**Figure S1. Flow chart of the study design**

**1999-2018 NHANES**

1999-2000 n=9,965 2001-2002 n=11,039

2003-2004 n=10,122 2005-2006 n=10,348

2007-2008 n=10,149 2009-2010 n=10,537

2011-2012 n=9,756 2013-2014 n=10,175

2015-2016 n=9,971 2017-2018 n=9,254

**46,582 were excluded**

<18 years n=42,112

Pregnant n=1,596

Missing follow-up data n=2,874

**54,734 were included**

**1999-2018 NHANES**

**n=101,316**

**3,482 were excluded**

Without total cholesterol or HDL n=3,462

Non-HDL>400 n=20

**51,252 were included**

**in the final analysis**

**Figure S2. Subgroup analyses of cancer and other-cause mortality risks in the ≤100mg/dL, >200mg/dL vs. 131-160mg/dL non-HDL-C group (n=51252)**

**
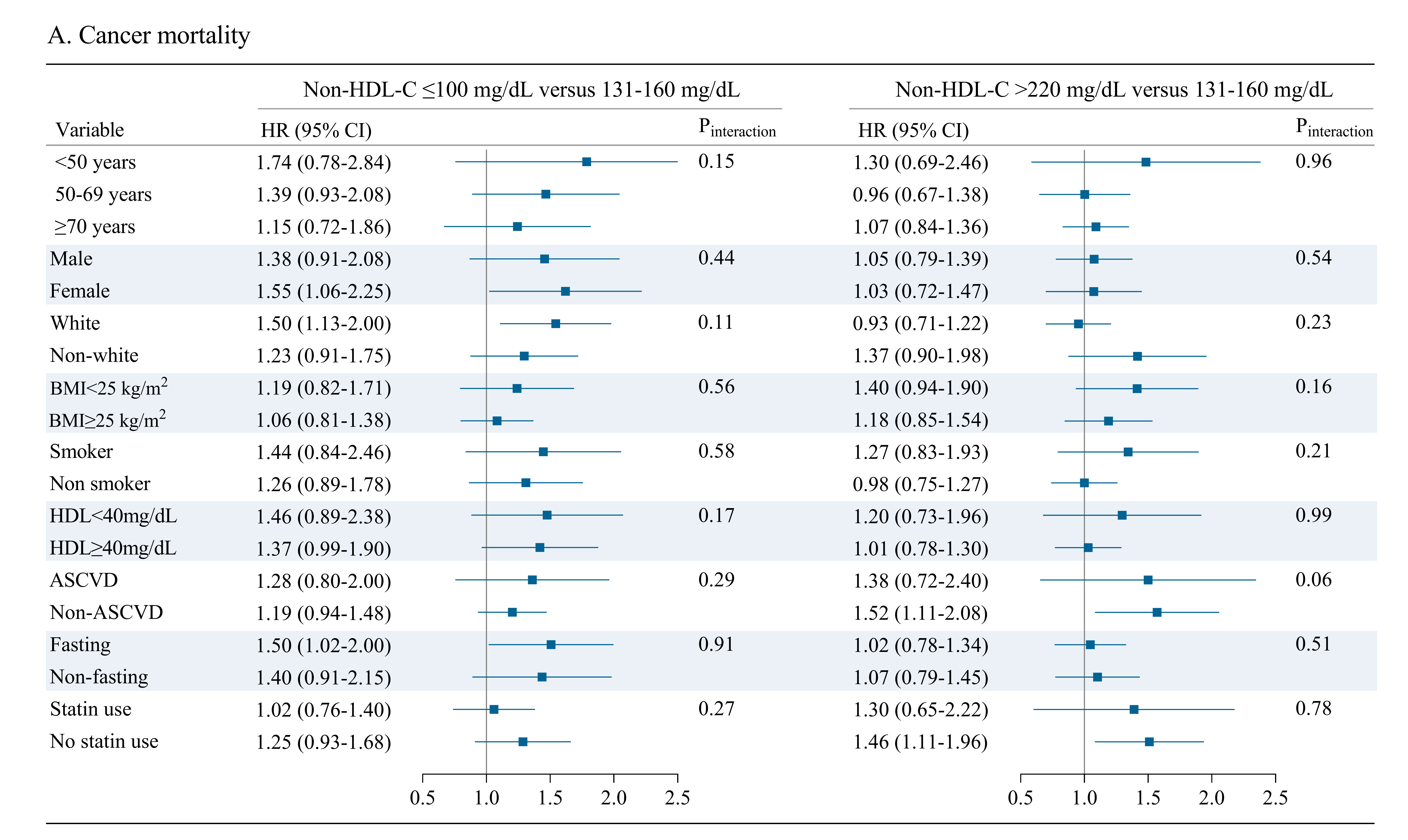
**

**
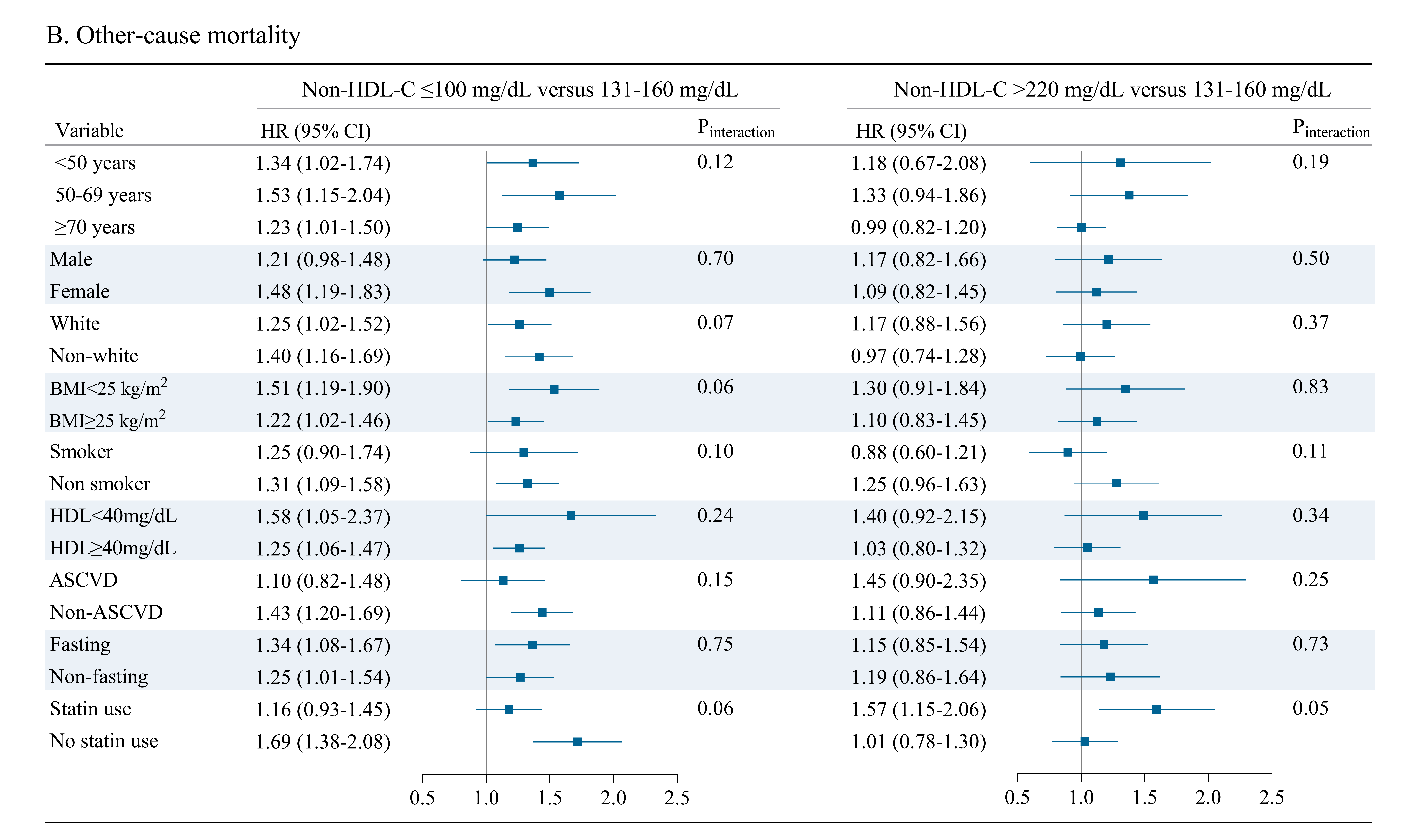
**

Adjusted for age, sex, ethnicity, systolic blood pressure, body mass index, HDL cholesterol, ASCVD, diabetes mellitus, COPD, renal dysfunction, cancer, fasting status. HDL, high-density lipoprotein. ASCVD, atherosclerotic cardiovascular disease. COPD, chronic obstructive pulmonary disease. Non-HDL-C, non high-density lipoprotein cholesterol. HDL, high-density lipoprotein. ASCVD, atherosclerotic cardiovascular disease. COPD, chronic obstructive pulmonary disease.

**Figure S3. All-cause and cause-specific mortality for continuous non-HDLC with additional adjustment for triglycerides (n=51252)**


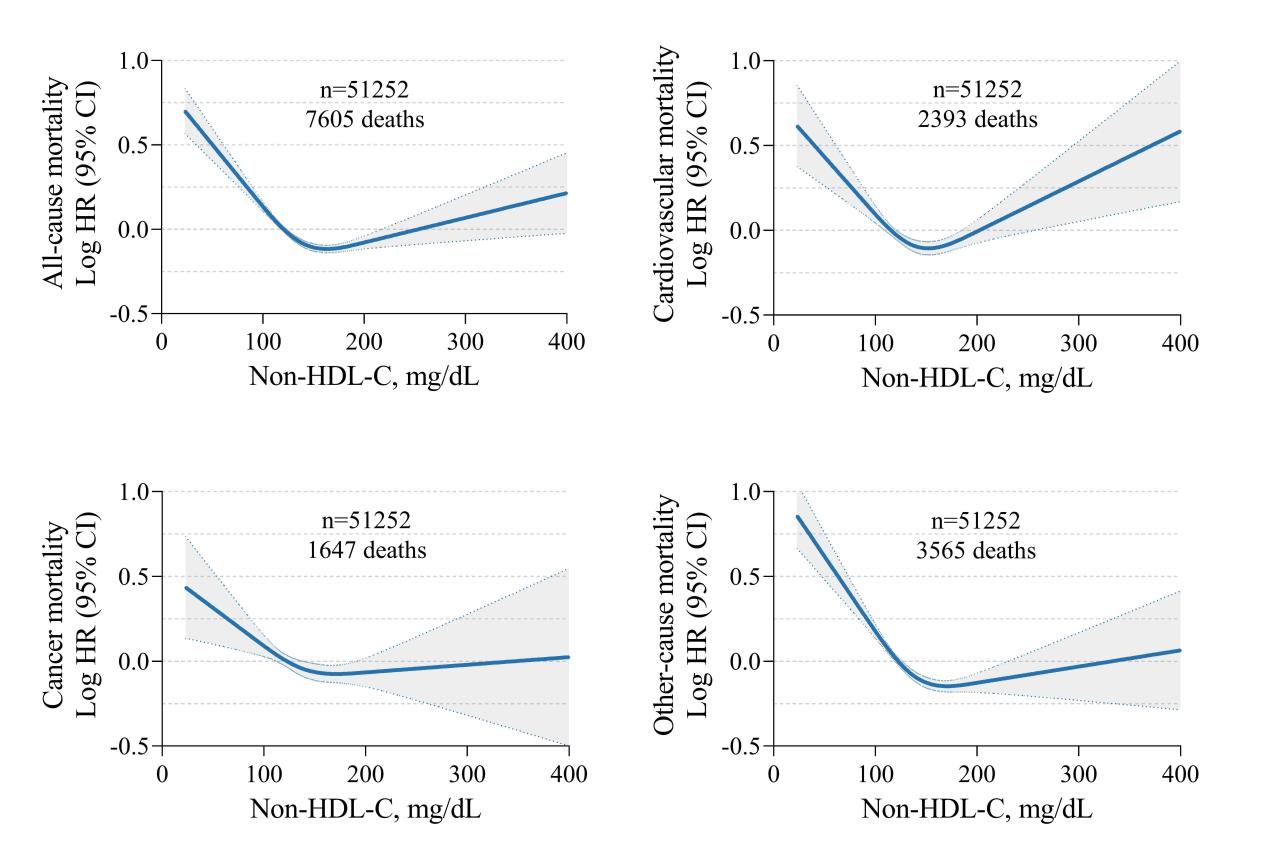


HRs (solid lines) and 95% CIs (shaded areas) were based on restricted cubic splines. HRs and 95% CIs were based on Cox proportional hazards model with non-HDL-C as a continuous variables. Adjusted for age, sex, ethnicity, systolic blood pressure, body mass index, **triglycerides**, HDL cholesterol, ASCVD, diabetes mellitus, COPD, renal dysfunction, cancer, fasting status. Non-HDL-C, non high-density lipoprotein cholesterol. HDL, high-density lipoprotein. ASCVD, atherosclerotic cardiovascular disease. COPD, chronic obstructive pulmonary disease.

**Figure S4. All-cause and cause-specific mortality for continuous non-HDL-C with additional adjustment for socioeconomic factors (n=51252)**

**
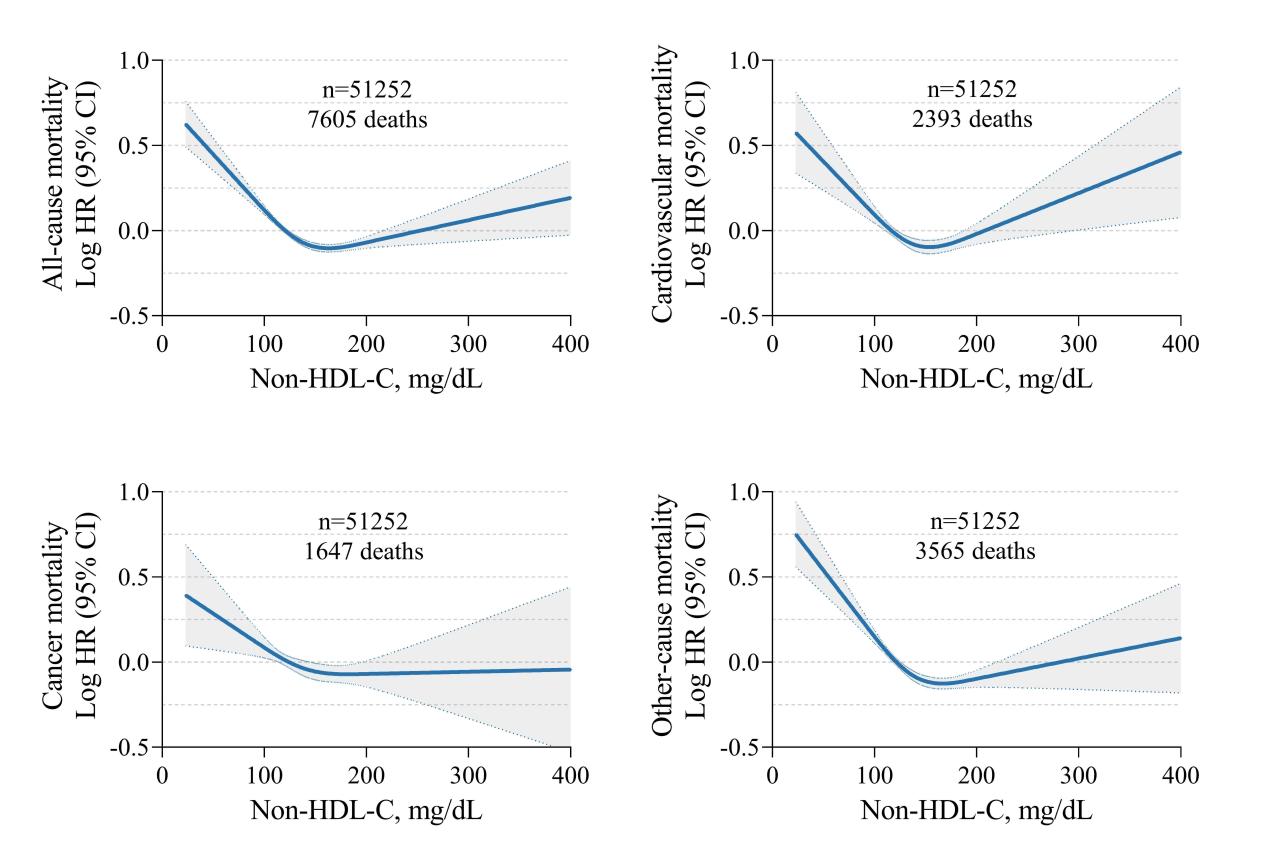
**

HRs (solid lines) and 95% CIs (shaded areas) were based on restricted cubic splines. HRs and 95% CIs were based on Cox proportional hazards model with non-HDL-C as a continuous variables. Adjusted for age, sex, ethnicity, systolic blood pressure, body mass index, HDL cholesterol, ASCVD, diabetes mellitus, COPD, renal dysfunction, cancer, fasting status, **education level, marital status, and poverty income ratio**. Non-HDL-C, non high-density lipoprotein cholesterol. HDL, high-density lipoprotein. ASCVD, atherosclerotic cardiovascular disease. COPD, chronic obstructive pulmonary disease.

**Figure S5. All-cause and cause-specific mortality for continuous non-HDL-C with additional adjustment for waist circumference (n=51252)**


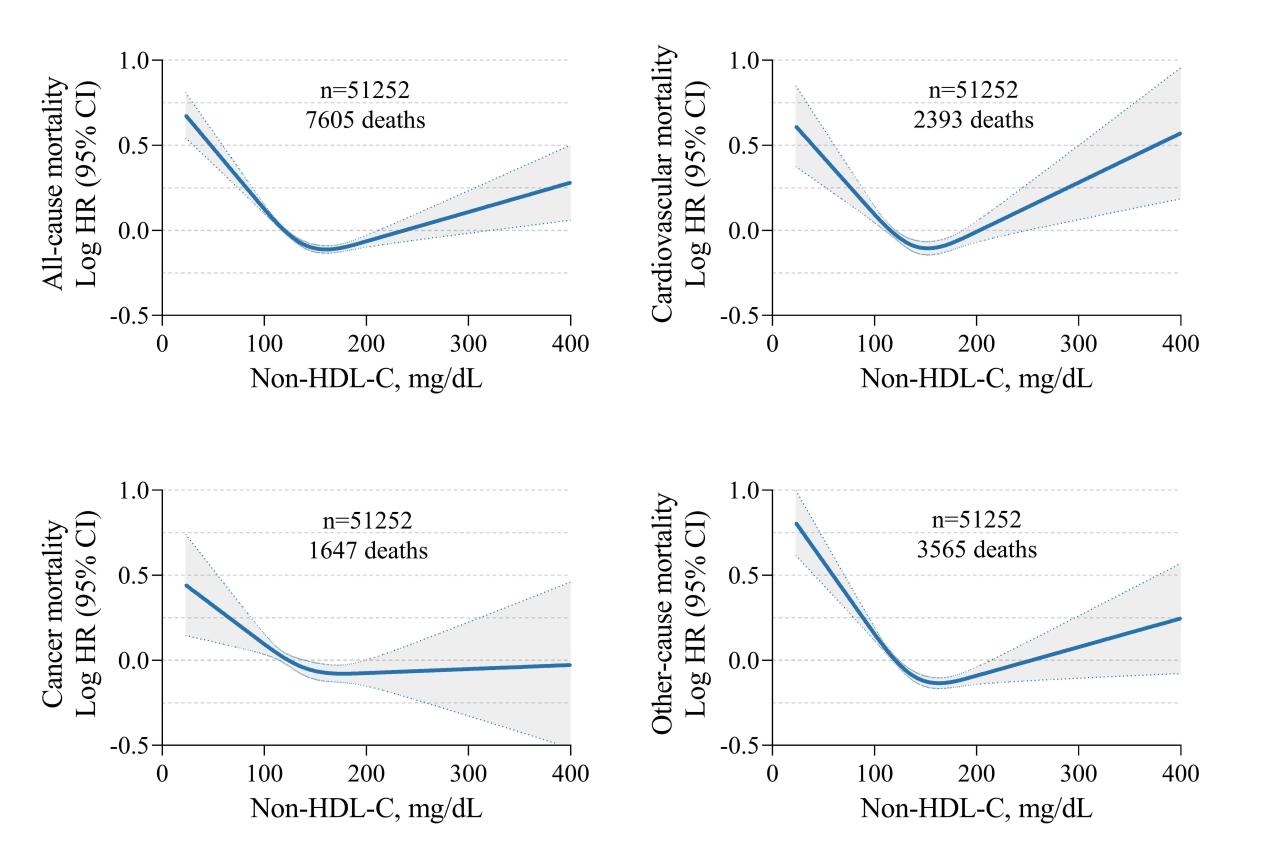


HRs (solid lines) and 95% CIs (shaded areas) were based on restricted cubic splines. HRs and 95% CIs were based on Cox proportional hazards model with non-HDL-C as a continuous variables. Adjusted for age, sex, ethnicity, systolic blood pressure, body mass index, **waist circumference**, HDL cholesterol, ASCVD, diabetes mellitus, COPD, renal dysfunction, cancer, fasting status. Non-HDL-C, non high-density lipoprotein cholesterol. HDL, high-density lipoprotein. ASCVD, atherosclerotic cardiovascular disease. COPD, chronic obstructive pulmonary disease.

**Figure S6. All-cause and cause-specific mortality for continuous non-HDL-C with exclusion of individuals with 1 year of follow-up (n=50791)**


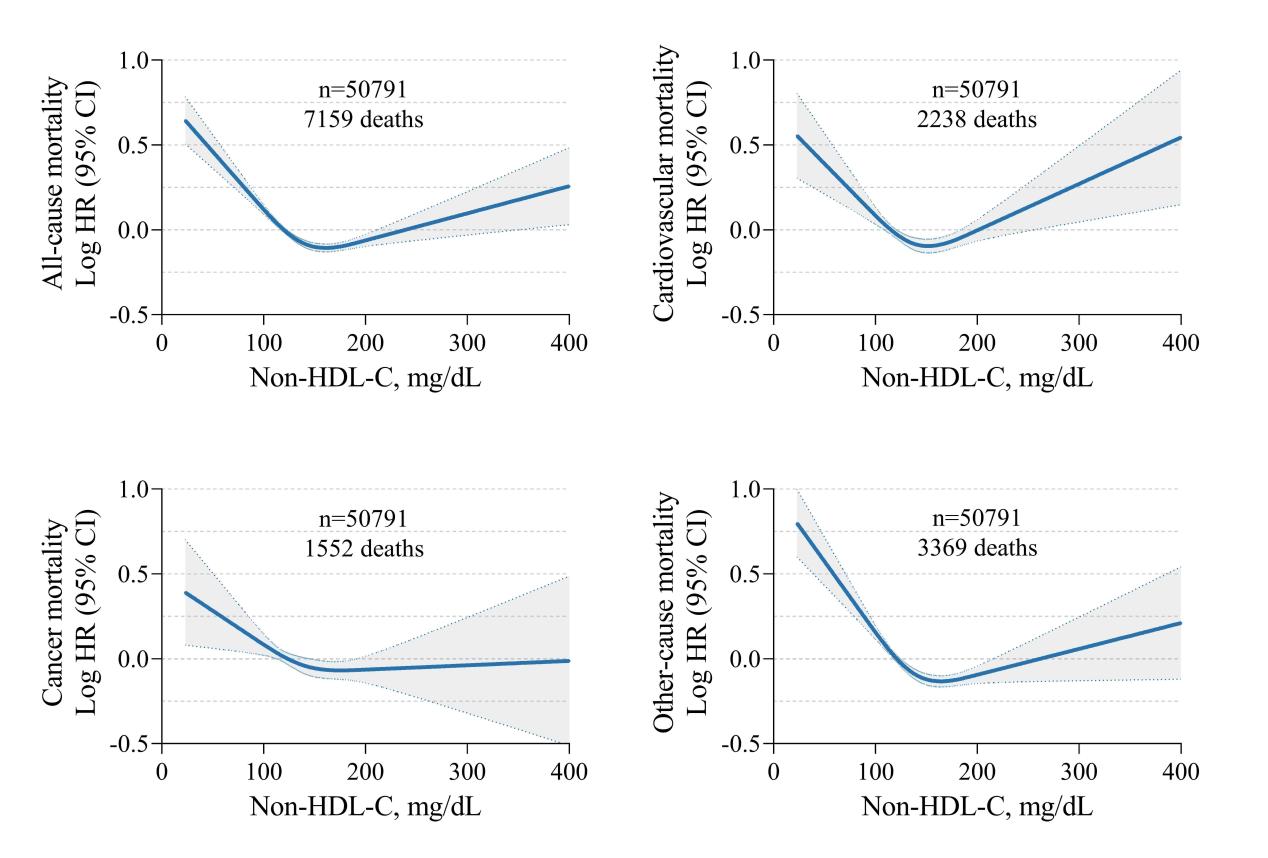


HRs (solid lines) and 95% CIs (shaded areas) were based on restricted cubic splines. HRs and 95% CIs were based on Cox proportional hazards model with non-HDL-C as a continuous variables. Adjusted for age, sex, ethnicity, systolic blood pressure, body mass index, HDL cholesterol, ASCVD, diabetes mellitus, COPD, renal dysfunction, cancer, fasting status. Non-HDL-C, non high-density lipoprotein cholesterol. HDL, high-density lipoprotein. ASCVD, atherosclerotic cardiovascular disease. COPD, chronic obstructive pulmonary disease.

**Figure S7. All-cause and cause-specific mortality for continuous non-HDL-C with exclusion of individuals with <2 years of follow-up (n=47864)**


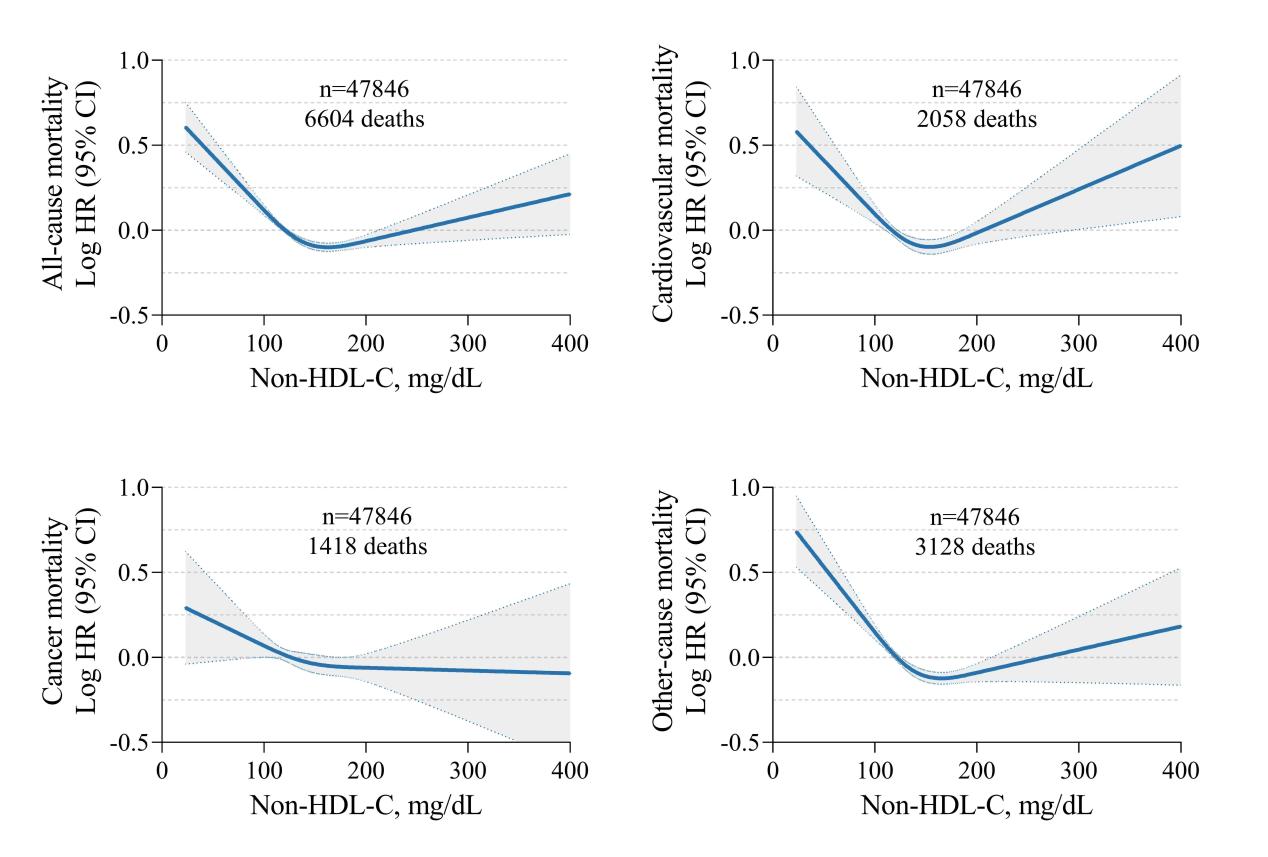


HRs (solid lines) and 95% CIs (shaded areas) were based on restricted cubic splines. HRs and 95% CIs were based on Cox proportional hazards model with non-HDL-C as a continuous variables. Adjusted for age, sex, ethnicity, systolic blood pressure, body mass index, HDL cholesterol, ASCVD, diabetes mellitus, COPD, renal dysfunction, cancer, fasting status. Non-HDL-C, non high-density lipoprotein cholesterol. HDL, high-density lipoprotein. ASCVD, atherosclerotic cardiovascular disease. COPD, chronic obstructive pulmonary disease.

**Figure S8. All-cause and cause-specific mortality risks in individuals with or without statin use**


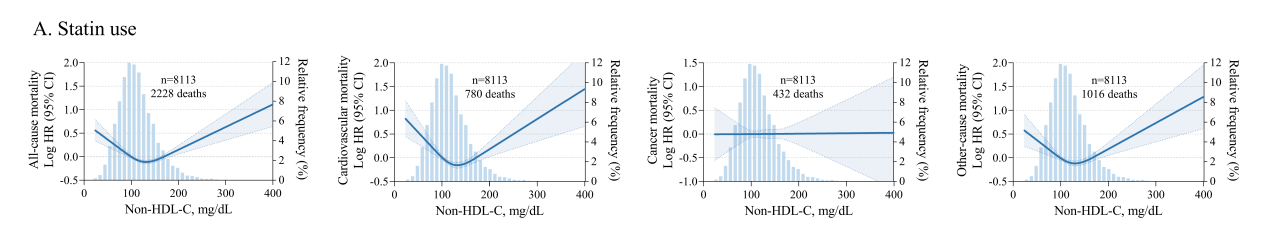


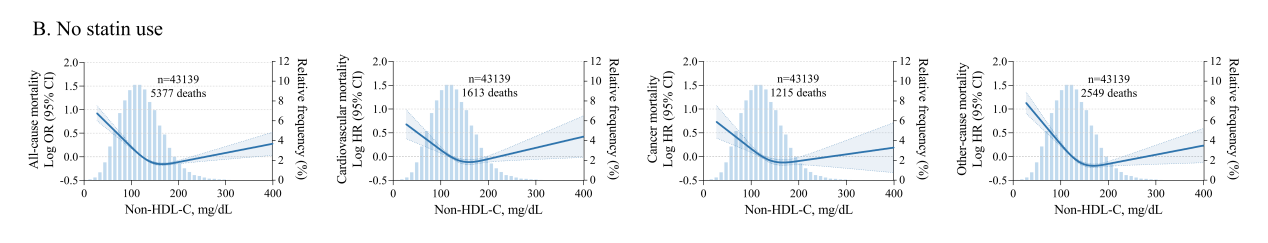


Associations between non-HDL-C and all-cause, cause-specific mortality in individuals with (A) or without statin use (B). HRs (solid lines) and 95% CIs (shaded areas) were based on restricted cubic splines. HRs and 95% CIs were based on Cox proportional hazards model with non-HDL-C as a continuous variables. The blue histograms show the distribution of non-HDL-C in the population. Adjustment variables include age, sex, ethnicity, systolic blood pressure, body mass index, HDL cholesterol, ASCVD, diabetes mellitus, COPD, renal dysfunction, cancer, fasting status. Non-HDL-C, non high-density lipoprotein cholesterol. HDL, high-density lipoprotein. ASCVD, atherosclerotic cardiovascular disease. COPD, chronic obstructive pulmonary disease.

**Figure S9. Subcategory of cause-specific mortality risks by non-HDL-C on continuous scales**


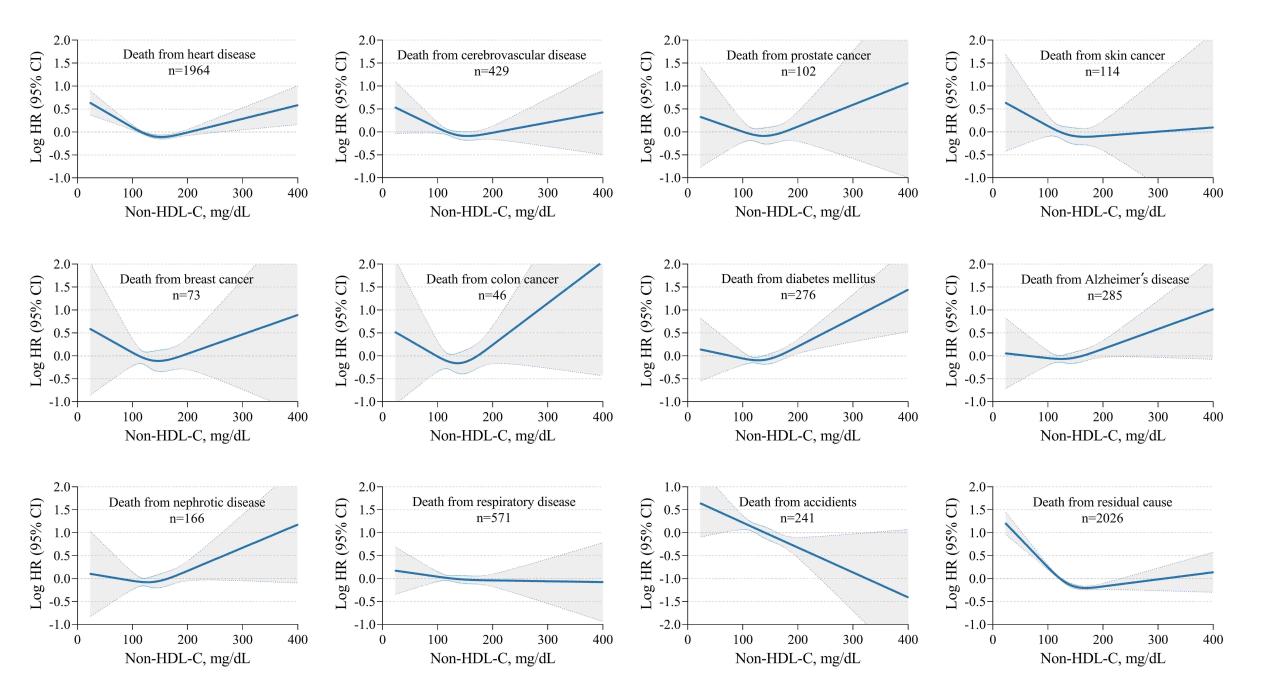


HRs (solid lines) and 95% CIs (shaded areas) are based on restricted cubic splines with Cox model. Adjusted for age, sex, ethnicity, systolic blood pressure, body mass index, HDL cholesterol, ASCVD, diabetes mellitus, COPD, renal dysfunction, cancer, fasting status. Skin cancer includes melanocytic carcinoma, non-melanocytic carcinoma, and other unknown skin cancer. HDL, high-density lipoprotein. Non-HDL-C, non high-density lipoprotein cholesterol. ASCVD, atherosclerotic cardiovascular disease. COPD, chronic obstructive pulmonary disease.

**Figure S10. All-cause and cause-specific mortality risks by age (<55, 55-65, and ≥65 years) and non-HDL-C**

**
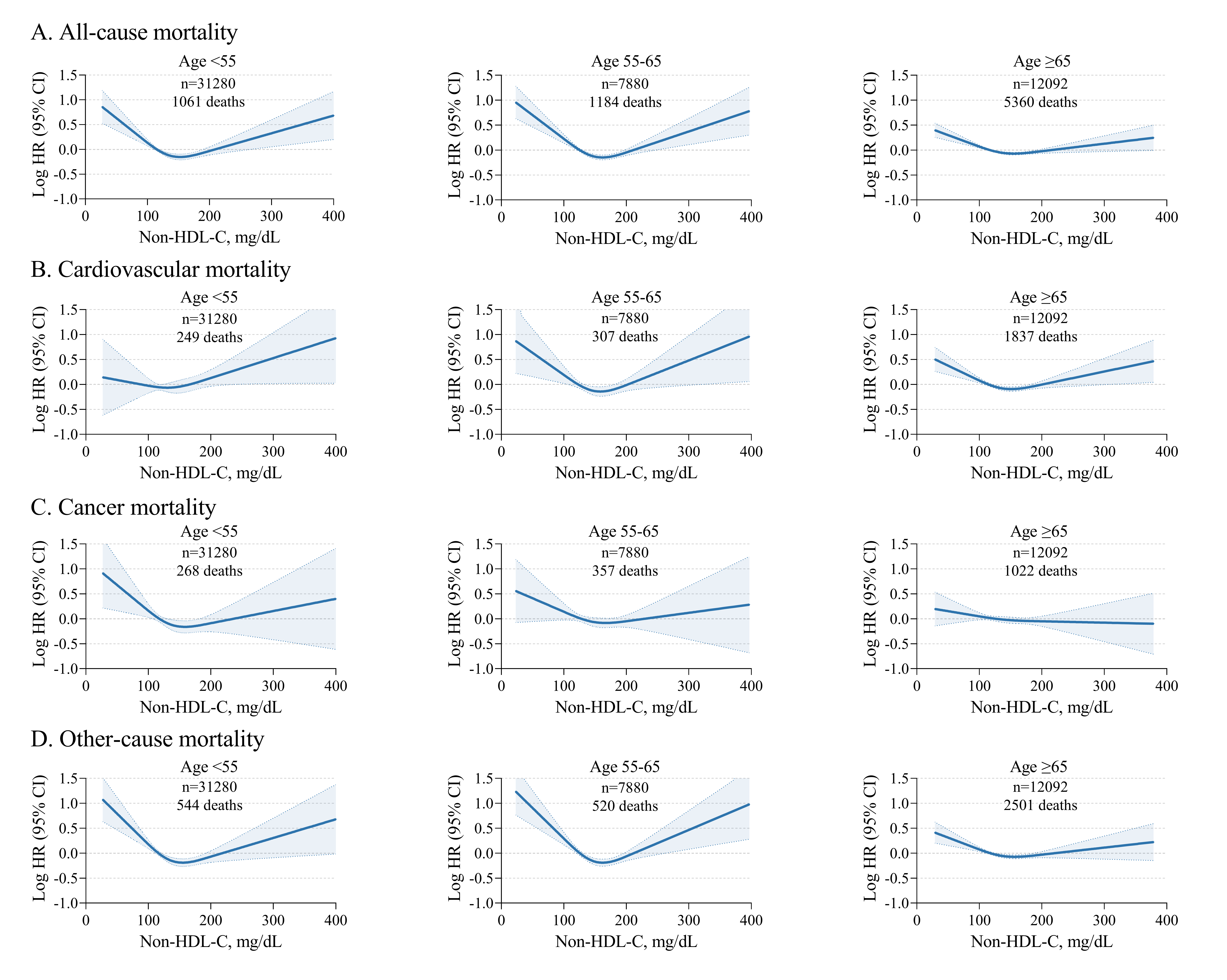
**

**Figure S11. All-cause and cause-specific mortality risks by age (<60, 60-80, and ≥80 years) and non-HDL-C**


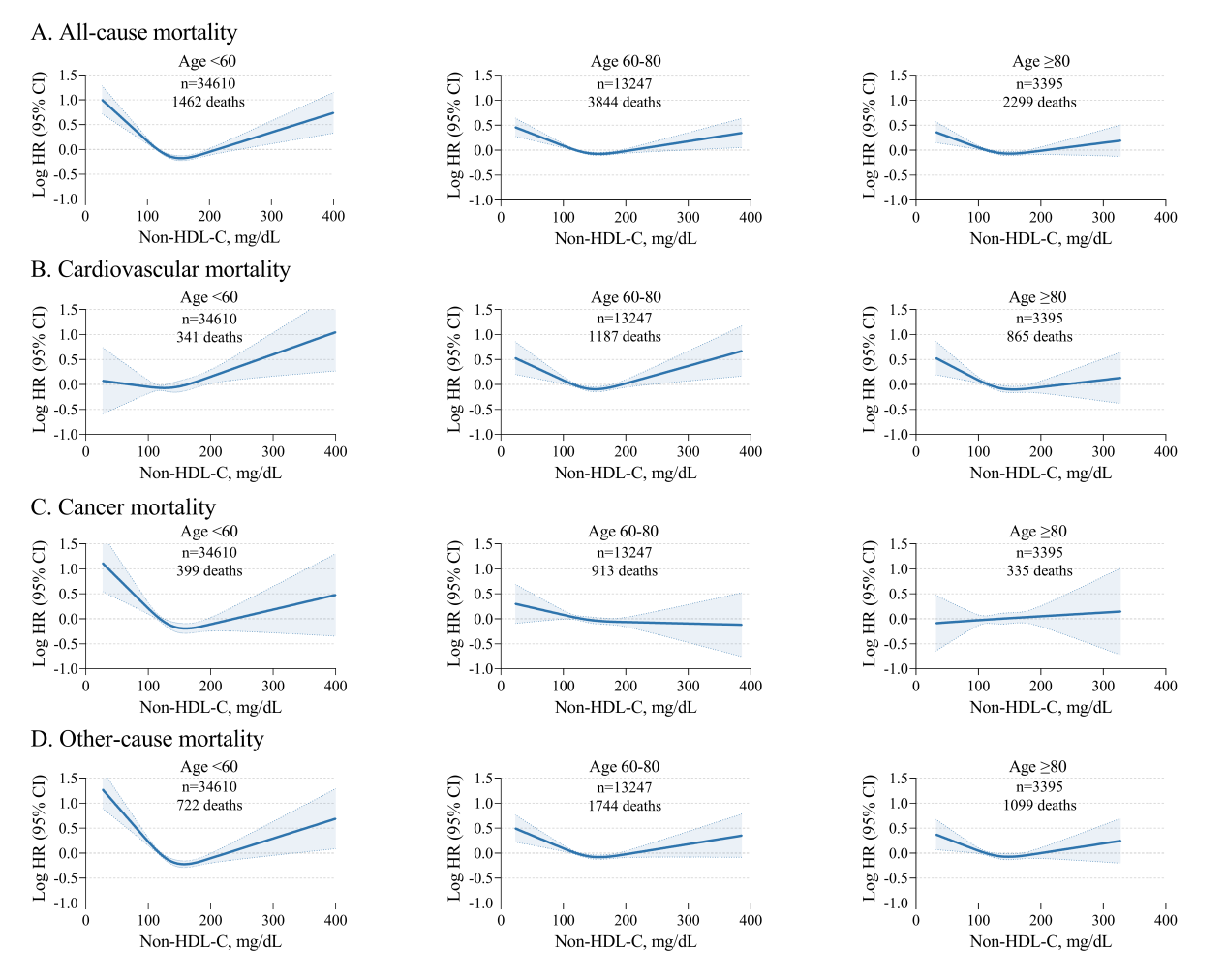

Supplement: Supplementary file 1 [file Table_1.docx]
